# Summertime Acute Heat Illness in U.S. Emergency Departments from 2006 through 2010: Analysis of a Nationally Representative Sample

**DOI:** 10.1289/ehp.1306796

**Published:** 2014-06-17

**Authors:** Jeremy J. Hess, Shubhayu Saha, George Luber

**Affiliations:** 1 Climate and Health Program, Division of Environmental Hazards and Health Effects, National Center for Environmental Health, Centers for Disease Control and Prevention, Atlanta, Georgia, USA; 2 Department of Emergency Medicine, Emory University School of Medicine, Atlanta, Georgia, USA; 3 Department of Environmental Health, Rollins School of Public Health at Emory University, Atlanta, Georgia, USA

## Abstract

Background: Patients with acute heat illness present primarily to emergency departments (EDs), yet little is known regarding these visits.

Objective: We aimed to describe acute heat illness visits to U.S. EDs from 2006 through 2010 and identify factors associated with hospital admission or with death in the ED.

Methods: We extracted ED case-level data from the Nationwide Emergency Department Sample (NEDS) for 2006–2010, defining cases as ED visits from May through September with any heat illness diagnosis (ICD-9-CM 992.0–992.9). We correlated visit rates and temperature anomalies, analyzed demographics and ED disposition, identified risk factors for adverse outcomes, and examined ED case fatality rates (CFR).

Results: There were 326,497 (95% CI: 308,372, 344,658) cases, with 287,875 (88.2%) treated and released, 38,392 (11.8%) admitted, and 230 (0.07%) died in the ED. Heat illness diagnoses were first-listed in 68%. 74.7% had heat exhaustion, 5.4% heat stroke. Visit rates were highly correlated with annual temperature anomalies (Pearson correlation coefficient 0.882, *p* = 0.005). Treat-and-release rates were highest for younger adults (26.2/100,000/year), whereas hospitalization and death-in-the-ED rates were highest for older adults (6.7 and 0.03/100,000/year, respectively); all rates were highest in rural areas. Heat stroke had an ED CFR of 99.4/10,000 (95% CI: 78.7, 120.1) visits and was diagnosed in 77.0% of deaths. Adjusted odds of hospital admission or death in the ED were higher among elders, males, urban and low-income residents, and those with chronic conditions.

Conclusions: Heat illness presented to the ED frequently, with highest rates in rural areas. Case definitions should include all diagnoses. Visit rates were correlated with temperature anomalies. Heat stroke had a high ED CFR. Males, elders, and the chronically ill were at greatest risk of admission or death in the ED. Chronic disease burden exponentially increased this risk.

Citation: Hess JJ, Saha S, Luber G. 2014. Summertime acute heat illness in U.S. emergency departments from 2006 through 2010: analysis of a nationally representative sample. Environ Health Perspect 122:1209–1215; http://dx.doi.org/10.1289/ehp.1306796

## Introduction

Heat has various effects on the human body, including several well-recognized acute heat illness syndromes (Kilbourne 1997), exacerbations of chronic lung and cardiovascular disease (Kovats and Hajat 2008), and multiple other syndromes associated with dehydration (Josseran et al. 2009). Acute heat illnesses are a subset of hyperthermia syndromes (Stitt 1979) resulting primarily from diminished heat dissipation capacity, classically from environmental exposure, although exertion may be a factor. Acute heat illness severity ranges from mild (e.g., heat rash, cramps, and fatigue) to moderate (e.g., heat syncope and heat exhaustion) to life-threatening (e.g., heat stroke). Heat stroke is defined as a core temperature above 40°C and evidence of neurological dysfunction (Knochel and Reed 1994). Mild to moderate heat illness is reversible with prompt treatment. Heat stroke, however, has a high case fatality rate, 51% at 28 days to 71% at 2 years in one cohort, and survivors have significant sequelae (Argaud et al. 2007). Although there is insufficient evidence surrounding the best resuscitative measures (Smith and Wallis 2005), outcomes are clearly improved by early intervention (Bouchama et al. 2007).

Although heat-related mortality is extensively studied, not as much is known about heat-related morbidity generally or acute heat illness specifically (Ye et al. 2012). In the United States, acute heat illness presents most commonly to the emergency department (ED), and > 80% of acute heat illness hospitalizations come through the ED—twice the proportion for other illnesses (Merrill et al. 2008). ED volume increases during periods of intense heat: A study of the 2006 California heat wave, for instance, found that the rate ratio for acute heat illness {defined as *International Classification of Diseases, 9th Revision, Clinical Modification* (ICD-9-CM) [Centers for Disease Control and Prevention (CDC) 2013] codes of 992} was 6.3 for ED visits and 10.2 for hospitalizations compared with the reference period (Knowlton et al. 2009). To date, however, studies of heat illness in the ED have been limited in scope, focusing on particular events, demographics, or subsets of ED visits.

There are several compelling reasons to examine acute heat illness in the ED. First, it is likely a good indicator of trends for other, more prevalent diseases affected by heat, as evidenced in the 2006 California heat wave (Knowlton et al. 2009). Second, many vulnerable groups use the ED disproportionately (Pitts et al. 2008), including elders, the poor, the chronically ill, and those with mental health concerns (Ishigami et al. 2008). Third, the ED is an appropriate setting for surveillance (Josseran et al. 2009) and tertiary prevention. Fourth, emergency physicians frequently clarify and advocate for appropriate preventive measures (Kellermann and Todd 1996). Fifth, ED heat illness visits may become more prevalent as the frequency of record heat increases (Meehl et al. 2009). In general, climate change has implications for heat illness as part of routine occupational and recreational activities (Maloney and Forbes 2011) and is likely to affect emergency providers disproportionately (Hess et al. 2009).

Despite the importance of the ED in managing acute heat illness, there are no comprehensive national estimates of ED visits for heat that include both fatal and nonfatal cases and are based on all ED diagnoses for a patient’s visit. Moreover, the available research does not clarify whether factors associated with presentation to the ED for heat illness are the same as those for heat-related deaths. Different risk profiles for morbidity and mortality based on disease pathogenesis (e.g., heat-related cerebrovascular injuries are more likely to be rapidly fatal than heat-related respiratory disease exacerbations) have been observed elsewhere (Kovats et al. 2004) and there may be implications for prevention programming and surveillance.

*Objectives*. We had three objectives with this analysis. The first was to estimate the burden of summertime acute heat illness using a nationally representative sample of ED patients to generate population-based rates for acute heat illness ED visits. The second was to characterize acute heat illness case profiles across time and different ED dispositions. Our third objective was to identify demographic factors and comorbid conditions associated with adverse outcomes of hospital admission or death in the ED among patients with acute heat illness.

## Methods

*Study design*. This study was an analysis of 5 recent years of data (2006–2010) from the Nationwide Emergency Department Sample (NEDS) of the Healthcare Cost and Utilization Project (HCUP) [Agency for Healthcare Research and Quality (AHRQ) 2014], an annual stratified sample of hospital-based EDs sponsored by the AHRQ (Steiner et al. 2002). The NEDS nationally representative database is constructed using a 20% stratified sample of U.S. hospital-based EDs from states contributing data to HCUP. Detailed information regarding sampling methods is available online at the AHRQ website (AHRQ 2011) and elsewhere (Owens et al. 2010). The NEDS has been used for multiple studies of various conditions seen commonly in EDs and is among the few nationally representative ED data sets available (Owens et al. 2010).

*Assessment of possible exposure bias in NEDS sample*. Our first step was to check for potential exposure bias related to the NEDS sampling scheme, because the NEDS database was constructed with data from select states (there were 24 partner states in 2006; 5 were added through 2009, and 28 participated in 2010; see Supplemental Material, Table S1, for additional details). We checked to see whether anomalously high temperatures in the NEDS states were similar to those in non-NEDS states over the study period, on the supposition that the prevalence of ED visits for acute heat illness in selected states could not be nationally representative if there were systematic differences in exposure. From the National Climatic Data Center (NCDC) website (NCDC 2013), we obtained historic temperature information for the contiguous 48 states. For each study year we compared the difference between average daily maximum temperature for 1 May through 30 September to the 30-year normal (1971–2000) for each state. We then compared the average differences in maximum temperature for the NEDS states with the non-NEDS states and tested whether these differences were statistically significant (see Supplemental Material, Table S2). The tests show that the temperature patterns were statistically different between the two groups only for the year 2009, with the anomaly in non-NEDS states greater than NEDS states.

*Data collection and management*. Our next step was to complete the NEDS data use agreements and download ED visit data from AHRQ. Data for national population estimates were also downloaded from the AHRQ website (AHRQ 2012). Insurance data were downloaded from the U.S. Census Bureau website (U.S. Census Bureau 2013). Data included demographics, insurance status (primary billed insurance), urban–rural designation of case’s county of residence [adapted from the CDC National Center for Health Statistics Urban-Rural Classification Scheme for Counties (CDC 2014)], ED diagnoses (first-listed and other diagnoses available in the record of ED visit), and disposition (treat-and-release or discharge, admission to the same hospital, transfer to another facility, and died in the ED). We analyzed the data using SAS version 9.3 (SAS Institute Inc., Cary, NC).

Our outcomes of interest were any ED diagnosis of acute heat illness as designated by the ICD-9-CM codes 992.0 to 992.9 and/or with an injury code (E-code) of E900 (accident or injury from exposure to excessive environmental heat); hospital admission; and death in the ED. To increase the likelihood that cases were representative of environmental heat exposure, we defined cases as patients presenting to the ED during summertime months (1 May through 30 September). We excluded cases with an e-code indicating heat exposure in a man-made environment (E900.1).

We used the Clinical Classifications Software (CCS) created by HCUP for ICD-9-CM (http://www.hcup-us.ahrq.gov/toolssoftware/ccs/ccs.jsp) to collapse the large array of diagnoses into a smaller number of clinically meaningful categories, and used these categories in our regression analyses.

HCUP also provides a binary Chronic Condition Indicator (CCI), which we used to classify ICD-9-CM diagnosis codes as chronic or not. We then categorized the number of chronic conditions and created an index of chronic disease burden (categories included one, two, three, and four or more). We used this index in a series of multiple regressions to evaluate the contribution of comorbid chronic disease on adjusted odds of hospital admission or death in the ED among all ED visits in the sample, among all cases except those with heat stroke, and among heat stroke cases.

As a secondary data analysis, the study was exempted from institutional review.

*Data analysis*. To pursue our first and second objectives we estimated total counts and population-based rates of ED visits for acute heat illness by ED disposition. We then disaggregated these counts based on *a*) whether or not heat illness was listed as the first diagnosis for the visit; *b*) year of visit and demographic and geographic factors; and *c*) different heat illness diagnoses. We used the statistical methodology prescribed by AHRQ (2012) to incorporate the complex survey design and use appropriate weights to generate national counts of ED episodes. We also used prescribed AHRQ methodology to estimate national rates for specific variables (AHRQ 2012). Because ambient temperature anomalies varied across time (see Supplemental Material Table S2), we used Spearman’s rho to evaluate the association between average annual temperature anomaly in NEDS states and annual ED heat illness visit rate.

To pursue our third objective, we conducted a series of regressions on the entire sample and on subsets defined by specific heat illness diagnoses. Because there was a gradient in severity of outcomes, we first considered ordinal logistic regression. We decided against that approach because model diagnostics indicated that the proportional odds assumption was violated. We also estimated a polytomous regression model with the three ED dispositions, but were circumspect of these results given the relatively small number of died-in-the-ED cases, so we present these results in Supplemental Material, Table S3. Our final approach was to perform logistic regression on a binary outcome variable created by combining the hospitalization and death-in-the-ED outcomes into one group. For the logistic regression we compared this composite outcome with the treat-and-release disposition. Independent variables included case-level demographic information, health insurance status, median income for case’s residential ZIP code, urban classification of county of residence, and CCS category of comorbid disease as described above.

Our last set of regressions used chronic disease burden category as described above as a predictor variable and examined adjusted odds of the composite outcome in the entire sample and in cases with heat stroke and any acute heat illness except heat stroke, controlling for the same variables described in the analyses above. Our hypothesis in this analysis was that adjusted odds of the composite outcome would be positively correlated with chronic disease burden.

To evaluate the ED case fatality rate (CFR) for heat stroke (the rate at which cases with heat stroke died in the ED), we compared ED CFR for heat stroke cases with the ED CFR for the cases with any heat illness diagnosis as well as the ED CFR for all ED visits from 2006 through 2010, stratified by CCS.

## Results

*Estimates of summertime acute heat illness ED visits*. Table 1 presents estimates of mean case counts and annualized population-based rates stratified by first-listed and secondary diagnosis and ED disposition. There were an estimated 326,497 [95% confidence interval (CI): 308,372, 344,658] cases in the study period (94% of all heat illness visits in the sample occurred between 1 May and 30 September). Dividing by the number of years in the study period yields an average of 65,299 ED visits for summertime acute heat illness per year—an average rate of 21.5 visits/100,000 population/year. According to estimates provided by HCUP (http://hcupnet.ahrq.gov/HCUPnet.jsp), there were 625 million ED visits in the United States from 2006 through 2010. Using this as the denominator and assuming no repeat visits for heat illness, this suggests an approximate average of 5/10,000 ED visits were for summertime heat illness during the study period.

**Table 1:** Counts and rates*^a^* (per 100,000 population per year) of acute heat illness (ICD-9-CM code 992.xx) with 95% CIs by first-listed and secondary diagnosis and ED disposition.*^b^*

ED disposition	First-listed diagnosis	Secondary diagnoses	Combined
Treat-and-release
*n*	202,829 (192,015–213,643)	85,046 (79,493–90,599)	287,875 (272,134–3,036,152)
Rate	13.3	5.6	18.9
Admit-to-hospital
*n*	18,811 (17,494–20,127)	19,581 (18,426–20,736)	38,392 (36,051–40,732)
Rate	1.2	1.3	2.5
Died-in-the-ED
*n*	99 (68–129)	131 (86–177)	230 (187–274)
Rate	0.007	0.009	0.015
Total
*n*	221,739 (209,577–233,899)	104,758 (98,055–111,512)	326,497 (308,372–344,658)
Rate	14.6	6.9	21.5
^***a***^Sum of total U.S. population for 2006 through 2010 used as denominator; population estimates used for each year were 309,349,689 (2010), 306,771,529 (2009), 304,093,966 (2008), 301,231,207 (2007), 298,379,912 (2006) (AHRQ 2012). ^***b***^ED visits between 1 May and 30 September in a calendar year.			

About 88% of cases were treated and released, 12% were admitted to the hospital, and a small number died in the ED (Table 1). Sixty-eight percent of the cases had a first-listed diagnosis of heat illness, and among them, 91% were treated and released. In the 32% of cases with a secondary diagnosis of heat illness, 81% were treated and released. The difference in percentage of treat-and-release disposition between cases with primary and secondary heat illness diagnoses was statistically significant (*p* < 0.001). However, the demographic, socioeconomic, insurance, and comorbid condition profiles of the two groups were not statistically different (results available on request).

*Acute heat illness case profiles across time and ED dispositions*. Table 2 shows univariate associations between demographic and other factors and ED disposition for all cases of acute heat illness–related ED visits from 1 May through 30 September during the study period. Both counts as well as rates per 100,000 population per year are presented. Missing data were rare; the most commonly missing data were for income (3% of the observations), rurality (1%), and type of health insurance (11%).

**Table 2:** Descriptive statistics [*n* (rate)] of patients with acute heat illness (ICD-9-CM codes 992.0–992.9) by ED disposition (*n* = 326,497).

Variable	Treat and release	Admit to hospital	Death in ED
Year
2006	61,182 (20.5)	8,214 (2.75)	99 (0.033)
2007	59,641 (19.8)	7,539 (2.5)	38 (0.013)
2008	48,668 (16)	6,416 (2.11)	27 (0.009)
2009	42,517 (13.9)	5,729 (1.87)	—^*a*^ (≤ 0.004)
2010	75,867 (24.5)	10,494 (3.39)	57 (0.018)
Age groups (years)
0–17	44,342 (12)	1,430 (0.39)	102 (0.028)
18–45	147,241 (26.2)	11,432 (2.03)	—^*a*^ (≤ 0.003)
46–65	67,363 (17.1)	12,634 (3.22)	69 (0.018)
> 65	28,918 (14.9)	12,896 (6.65)	59 (0.031)
Sex
Male	197,043 (25.5)	28,591 (3.7)	186 (0.024)
Female	90,676 (12.1)	9,801 (1.31)	45 (0.006)
Hospital region
Northeast	32,130 (11.7)	3,997 (1.46)	19 (0.007)
Midwest	64,701 (19.5)	7,365 (2.21)	14 (0.004)
South	146,630 (26.2)	19,406 (3.46)	80 (0.014)
West	44,414 (12.6)	7,623 (2.16)	117 (0.033)
Median ZIP code income (quartile)
Lowest	91,390 (23.9)	13,372 (3.5)	103 (0.027)
Second	81,860 (21.6)	9,957 (2.63)	55 (0.014)
Third	61,171 (16.2)	7,613 (2.02)	30 (0.008)
Highest	45,174 (11.9)	5,656 (1.49)	23 (0.006)
Urban–rural classification
Large metropolitan	112,067 (13.8)	17,546 (2.16)	114 (0.014)
Medium/small metropolitan	99,258 (21.9)	12,011 (2.65)	60 (0.013)
Micropolitan	43,781 (29)	4,948 (3.27)	30 (0.02)
Rural	29,862 (29.7)	3,340 (3.32)	18 (0.018)
Payment source^*b*^
Medicare	38,427 (17.7)	14,950 (6.88)	75 (0.035)
Medicaid	35,713 (16.8)	3,581 (1.68)	70 (0.033)
Private insurance	115,112 (11.5)	10,058 (1)	16 (0.002)
Uninsured	67,062 (28.8)	6,058 (2.6)	48 (0.021)
Total number of observations under each category may be different due to missing data. Rates are per 100,000 population per year; denominators were obtained from AHRQ (2012). Data were pooled for 2006–2010; ED visits between 1 May and 30 September were included, and sample weights were used. ^***a***^Counts ≤ 10 were suppressed per AHRQ guidance. ^***b***^Health insurance coverage status 2006–2010 obtained from the U.S. Census Bureau (2013).			

**Figure 1:**
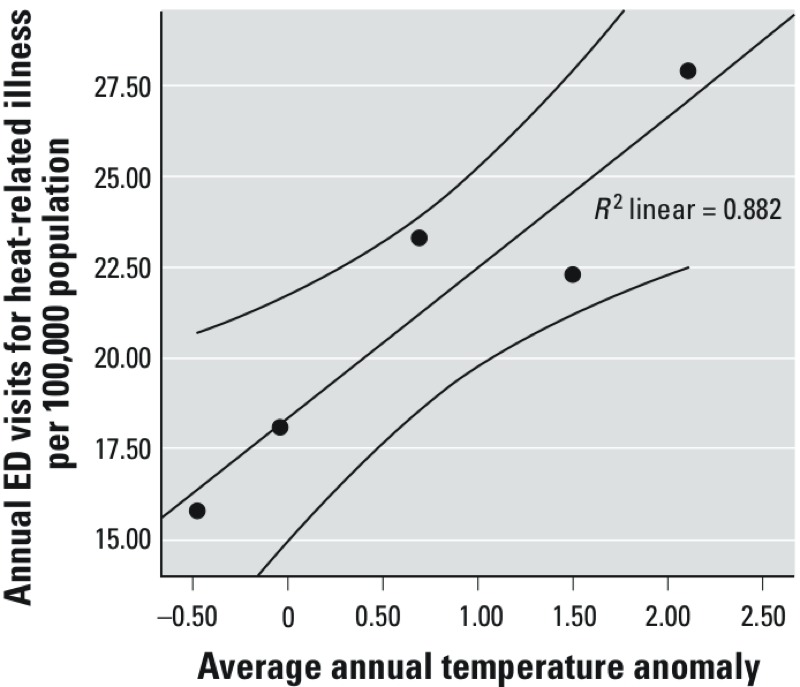
Annual population-based rates of ED visits for heat illness by average annual temperature anomalies for NEDS states. A trendline with 95% CI is included for reference. The Pearson correlation coefficient is 0.882, *p *= 0.005.

Annual counts and rates showed significant interannual variation, with highest counts and rates (apart from the rate of death in the ED) in 2010. Annual temperature anomalies for NEDS states are shown in Supplemental Material, Table S2. As shown in Figure 1, there is a significant correlation between annual temperature anomalies and annual population-based rate for ED heat illness visits.

In regard to demographic variables, the 18–45 age group had the highest treat-and-release rate, whereas the ≥ 65 age group had the highest rates of hospital admission and death in the ED. Males were more likely than females to have heat illness across all dispositions. The highest rates for treat-and-release and hospital admissions were in the South; the highest rate of death in the ED was in the West. Across all ED dispositions, the highest rates were found among people in the bottom income quartile. Areas designated as rural and micropolitan had higher rates across all ED dispositions compared with the areas designated as metropolitan. With respect to health insurance, the uninsured population had the highest rate among the treat-and-release visits, whereas cases with Medicare had the highest rates of hospital admission and death in the ED.

*Factors associated with adverse outcomes of hospital admission or death in the ED*. Counts of heat illness diagnoses stratified by ED disposition are presented in Table 3. Of all cases, 74.7% were diagnosed with heat exhaustion (ICD-9-CM codes 992.3–992.5), 5.4% with heat stroke (code 992.0) and 3.7% with nonspecific heat illness (code 992.9). Among those with heat stroke, 62.0% of cases were either admitted (28.3% of all admissions) or died in the ED (77.0% of all death-in-the-ED cases). The risk of fatality was much lower among heat exhaustion cases, 90.6% of which were treated and released when considered altogether.

**Table 3:** Counts of acute heat illness diagnoses (ICD9 codes 992.0–992.9) by emergency department (ED) disposition with row and column percents.

ICD-9-CM heat illness diagnosis	Treat and release			Admit to hospital			Death in ED		
	*n*	Row %^*a*^	Column%^*b*^	*n*	Row %	Column %	*n*	Row %	Column %
992.0–Heat stroke	6,889	38	2	11,059	61	28	178	1	77
992.1–Heat syncope	12,812	86	4	2,089	14	5	—^*c*^	≤ 2	≤ 4
992.2–Heat cramps	17,592	98	6	373	2	1	—^*c*^	≤ 2	≤ 4
992.3–Heat exhaustion (anhydrotic)	10,841	79	4	2,899	21	7	—^*c*^	≤ 2	≤ 4
992.4–Heat exhaustion (salt depletion)	1,252	67	0	623	33	2	—^*c*^	≤ 2	≤ 4
992.5–Heat exhaustion (unspecified)	213,659	91	73	19,894	9	51	—^*c*^	≤ 2	≤ 4
992.6–Heat fatigue	2,600	95	1	130	5	0	—^*c*^	0	≤ 4
992.7–Heat edema	629	99	0	—^*c*^	≤ 1	0	—^*c*^	≤ 2	≤ 4
992.8–Other specified heat effects	16,135	91	5	1,585	9	4	11	0	5
992.9–Unspecified	11,848	96	4	410	3	1	26	0	11
Data were pooled for 2006–2010; ED visits between 1 May and 30 September were included; sample weights were used. Patients could have more than one diagnosis of acute heat illness during a visit. Total diagnoses count therefore is greater than the number of patients. ^***a***^For each specific heat diagnosis, Row % refers to the percentage of patients with different ED disposition. ^***b***^For each ED disposition, Column % refers to the percentage of patients with different heat diagnoses. ^***c***^Counts ≤ 10 were suppressed per AHRQ guidance.									

Table 4 presents results from the logistic regression examining factors associated with the composite adverse outcome (hospital admission or death in the ED) among all cases. Adjusted odds ratios of hospitalization or death in the ED relative to treat-and-release are presented as the model controls for a wide range of factors—demographic, geographic, rural–urban indicator, health insurance type, chronic conditions, and diagnosis of heat illness. The > 65 group, males, residents of large metropolitan areas, the uninsured, residents of ZIP codes with low median household incomes, and cases with heat stroke had higher odds of hospitalization or death in the ED than treatment-and-release. There was relatively little meaningful significant regional or interannual variation compared with the significant rate differences in Table 2. Adjusted odds of hospital admission or death in the ED were increased by all chronic conditions in some cases substantially: Chronic hematologic conditions increased adjusted odds 9-fold.

**Table 4:** Logistic regression comparing odds ratios (ORs) of hospital admission/death among patients with heat stroke (ICD-9-CM code 992.0) and other acute heat illness diagnoses (ICD-9-CM codes 992.1–992.9) (*n* = 282,743).

Variable	OR (95% CI)
Age group (years)
0–17	0.4 (0.37, 0.43)
18–45	0.45 (0.42, 0.47)
46–65	0.59 (0.56, 0.62)
> 65 (referent)	1
Urban–rural classification
Large metropolitan	1.36 (1.29, 1.44)
Medium/small metropolitan	0.96 (0.91, 1.02)
Micropolitan	0.93 (0.87, 0.99)
Rural (referent)	1
Health insurance
Medicare	1.01 (0.96, 1.07)
Medicaid	0.88 (0.84, 0.93)
Private	0.94 (0.9, 0.98)
Uninsured (referent)	1
Median income for ZIP code
Highest quartile	0.76 (0.72, 0.8)
Third quartile	0.75 (0.72, 0.79)
Second quartile	0.83 (0.8, 0.86)
Lowest quartile (referent)	1
Geographic regions
Midwest	0.86 (0.82, 0.91)
South	0.92 (0.88, 0.97)
West	1 (0.94, 1.06)
Northeast (referent)	1
Sex
Male	1.64 (1.59, 1.7)
Female (referent)	1
Associated chronic conditions
Blood	9.05 (8.45, 9.69)
Circulatory	2.44 (2.36, 2.53)
Digestive	2.14 (2.02, 2.26)
Endocrine	2.85 (2.76, 2.95)
Genito-urinary	3.48 (3.25, 3.72)
Mental	2.75 (2.66, 2.84)
Muscular	2.43 (2.28, 2.58)
Nervous	3.22 (3.05, 3.39)
Respiratory	1.92 (1.83, 2.02)
Cancer	2.66 (2.3, 3.07)
No comorbid conditions (referent)	1
Heat diagnoses
Heat stroke (992.0)	23.86 (22.35, 25.46)
Heat syncope (992.1)	2.81 (2.6, 3.04)
Heat exhaustion (992.3–992.5)	2.03 (1.93, 2.14)
Other heat diagnosis (referent)	1
Year
2010	0.73 (0.7, 0.76)
2009	0.68 (0.65, 0.72)
2008	0.79 (0.76, 0.83)
2007	0.87 (0.83, 0.91)
2006	1
Data were pooled for 2006–2010; ED visits occurred from 1 May through 30 September; sample weights were used. The number of cases is smaller compared with those in Table 3 because some explanatory variables had missing variables.	

Although the small number of died-in-the-ED cases make us circumspect, we present the results of the polytomous logistic regression in Supplemental Material, Table S3. In this analysis, adjusted odds of death in the ED were highest for cases 0–17 years of age, males, Midwestern residents, residents of low-income ZIP codes, those with Medicaid, and those with chronic circulatory conditions.

Because the adjusted odds ratio of hospitalization or death in the ED among heat stroke cases was 23.86 (95% CI: 22.35, 25.46) (Table 4), we separately analyzed the possibility that these risk factors were different among this subgroup (see Supplemental Material, Tables S4 and S5). Among heat stroke cases, younger patients had higher adjusted odds of the composite outcome than did non–heat stroke cases; adjusted odds were also higher for heat stroke cases in large metropolitan areas, though the observed urban–rural gradient was the same. In both groups, cases from the Midwest had the lowest adjusted odds, though the difference was more marked for heat stroke cases. Other trends were similar between the two groups.

Table 5 shows the results of the ED CFR analysis. The CFR for all acute heat illness diagnoses is comparable to those of the other CCS groups. The CFR for heat stroke, 99.4/10,000 (95% CI: 78.7, 120.1) ED visits, is significantly higher than the other groups, the highest of which is 50.7/10,000 for circulatory disease.

**Table 5:** Comparing fatalities per 10,000 ED visits for acute heat illness and other illnesses based on HCUP CCS (pooled data from 2006–2010 from the entire NEDS sample).

HCUP clinical classification	Rate per 10,000 ED visits (95% CI)
Infectious and parasitic disease	7 (6.5, 7.4)
Neoplasms	29.3 (27.9, 30.6)
Endocrine, nutritional, metabolic diseases and immunity disorders	15.9 (15.1, 16.7)
Diseases of blood and blood-forming organs	9.1 (8.6, 9.6)
Mental illness	7.1 (6.7, 7.5)
Diseases of nervous system and sense organs	5.8 (5.5, 6.2)
Diseases of circulatory system	50.7 (48.5, 52.9)
Diseases of respiratory system	13 (12.3, 13.6)
Diseases of digestive system	5.8 (5.5, 6.1)
Diseases of genitourinary system	8.7 (8.2, 9.2)
Complications of pregnancy, childbirth and puerperium	0.7 (0.5, 0.9)
Diseases of skin and subcutaneous tissue	1.9 (1.8, 2.1)
Diseases of musculoskeletal and connective tissue	3.5 (3.2, 3.7)
Congenital anomalies	12 (11.3, 12.6)
Certain conditions originating in perinatal period	18.6 (17.4, 19.7)
All acute heat illness diagnoses	8.9 (7.8, 10)
Heat stroke	99.4 (78.7, 120.1)

**Figure 2:**
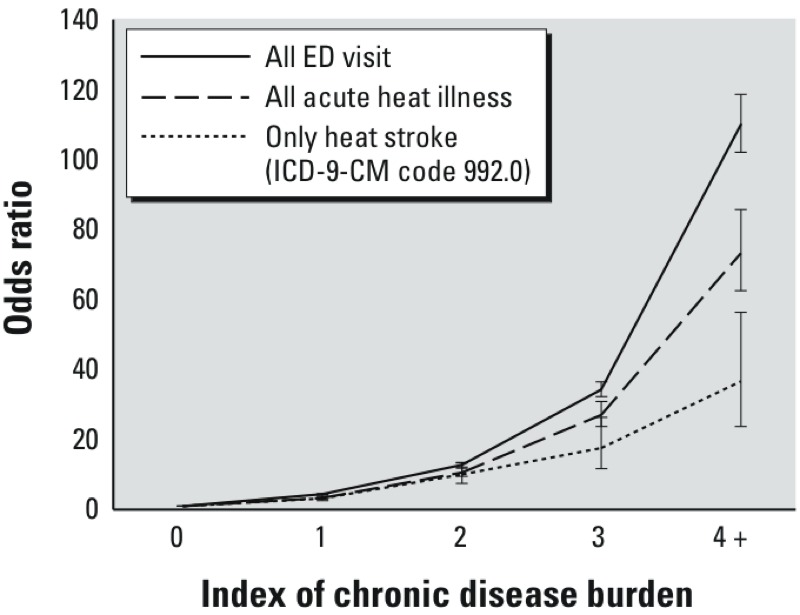
Adjusted odds ratios of hospital admission or death in the ED for different degrees of chronic disease burden, stratified by all ED visits, all acute heat illness, and only heat stroke visit (ICD-9-CM code 992.0). The index was calculated based on combining the CCS and CCI information provided in HCUP.

Figure 2 shows the results of the logistic regression analyses examining the association between the index of chronic disease burden and adjusted odds of the composite outcome (hospital admission or death) for three categories of ED patients: those with any heat illness except heat stroke, those with heat stroke, and all ED visits. In each analysis we found a sharp increase in the relationship between chronic conditions and adjusted odds of the composite outcome. The curve slopes varied, and were lowest for heat stroke cases and highest for all ED visits. Put differently, higher chronic disease burden was more strongly associated with odds of hospitalization or death in the ED for all ED visits than for heat stroke cases.

## Discussion

Our findings contribute to the burgeoning literature on heat and morbidity generally and, in particular, ED visits for acute heat illness. Our analysis suggests that, for the years studied, the NEDS is likely to provide reasonable national estimates of ED visits for summertime acute heat illness. Our results also suggest that acute heat illness is a substantial concern, and that heat exhaustion is the most common presentation. Our findings provide new evidence regarding the relatively high rates of acute heat illness in rural populations, the high ED CFR of heat stroke, and the dramatic role of age and chronic conditions on the likelihood of hospital admission or death in the ED. Our results also suggest that odds of death in the ED are highest among the young, the poor, Midwesterners, and males, though this requires further study. Finally, our results also appear to capture interannual variability in dose response, with higher visit rates in anomalously warm years (Figure 1; see also Supplemental Material, Table S2).

Our estimates are for acute heat illness only and not for associated conditions whose prevalence may be affected by heat exposure (i.e., the broader category of heat-associated illness). Although incidence of acute heat illness can rise dramatically during extreme heat events, incidence of several heat-associated illnesses also rises, and acute heat illness is a relatively small proportion of the total increased disease burden. In the 2006 California heat wave, for instance, acute heat illness cases made up only 13.2% of the excess ED visits (Knowlton et al. 2009). There was increased incidence in other disease categories, particularly electrolyte imbalance, nephritis, and renal failure, exclusive of an associated acute heat illness diagnosis. Thus, if patterns observed during heat waves can be applied to other summertime periods, our estimates likely represent only a fraction of the total burden of heat-related ED visits. Without exposure data, however, we cannot assess the validity of this claim.

The lack of exposure and other data also complicate interpretation of some of our other findings. The regional differences in died-in-the-ED rates among patients with heat illness are one example, and may relate to regional differences in exposure, illness severity, or admission practices, or to variability resulting from the relatively small sample size. The relatively high rate of heat illness in rural areas, which may reflect differences in occupational exposures in certain settings or to differential prevalence in air conditioning availability and usage, is another example.

There is only one prior national estimate of the incidence of ED visits for acute heat illness against which we can compare our findings (Sanchez et al. 2010); our estimates of heat exhaustion incidence, male predominance, and the age distribution of heat illness cases are all relatively consistent with theirs. The main difference is the overall incidence of heat illness among ED patients. Sanchez et al. (2010) estimated that there were 20,775 heat illness ED visits a year from 2001 through 2004, a third of our estimate for years 2006–2010. Part of the difference can be attributed to differences in case definition, because cases in that study were nonfatal visits with documented environmental heat exposure and first-listed diagnosis of heat injury. In our sample, limiting the case definition to nonfatal cases with first-listed diagnosis of heat illness (Table 1) yielded 221,640 cases for the study period or an average of 44,328 such cases annually—roughly double the estimate of Sanchez et al. (2010). Other possible causes for this difference are unclear. There may be bias in either or both of the estimates, or differential exposure among the sampled populations. Although temperatures have been gradually warming during this time, there has not been a significant difference in the frequency of heat waves between the two study periods (U.S. Environmental Protection Agency 2010). Regional temperature differences would presumably have a larger impact on estimates using the National Electronic Injury Surveillance System–All Injury Program (NEISS-AIP; http://www.healthindicators.gov/Resources/DataSources/NEISS-AIP_88/Profile), the data Sanchez et al. used, which collects data from 66 hospitals, than the NEDS, which samples roughly 1,000; but because investigators are blinded to hospital identities, it is difficult to assess this explanation. Finally, the other study’s strict inclusion criteria surely play some role and likely resulted in an underestimate, as the authors note.

We can also compare a subset of our findings with another national estimate of rates of ED visits for hyperthermia among Medicare beneficiaries in 2004–2005 (Noe et al. 2012). Here, too, our estimate is higher. They estimate a rate of 12.0 ED hyperthermia visits/100,000 population/year and 5.3 hospital admissions (not all from the ED) for heat illness/100,000 population/year with an admission proportion (the proportion of visits that result in hospital admission) of 11%. For cases with Medicare we estimate a rate of 24.6 ED visits/100,000 population/year, an admission rate from the ED of 6.9/100,000 population/year, and an admission proportion of 28.0% (Table 2). The differences are most likely attributable to inclusion criteria: Noe et al. (2012) included only cases with a first-listed diagnosis of hyperthermia, whereas we included cases if acute heat illness was one of any listed. Heat illness was listed first in 67.9% of our cases (Table 1), and this could account for the bulk of the difference, highlighting the importance of including secondary diagnoses in epidemiological studies of ED visits for heat illness. The remainder of the difference may result from interannual variability in visit rates and differences in the databases used for the respective analyses.

As might be expected, our findings suggest that heat illness is more prevalent among young adults and that hospitalization is more prevalent among older adults. Perhaps surprisingly, death in the ED is equally prevalent in the young and in elders. Differences in exposure and/or susceptibility may be responsible for these patterns. Younger people are presumably more exposed through occupational and recreational activities and have greater physiologic resilience, suggesting that a larger proportion of their heat illness presentations are less severe. This could, paradoxically, be the explanation for the relatively high prevalence of death in the ED among cases 0–17 years of age, in the event that younger people are able to compensate in the earlier stages of heat illness and present later in the illness course when treatment is less effective. That elders are more likely to be admitted to the hospital is potentially a function of higher exposure, to the extent that elders may have less capacity to recognize and reverse heat illness at earlier stages, or it may be related to their greater burden of underlying chronic disease. The higher prevalence of death in the ED among elders is presumably driven by physical frailty and underlying comorbid disease. Our data (Table 4) do demonstrate an association between chronic disease burden and complications of heat illness, suggesting that physiologic reserve is at least partially responsible, but this explanation is not exclusive.

That increasing chronic disease burden is associated with increased adjusted odds of hospital admission or death in the ED is unsurprising. What is perhaps more surprising is that the relative increase in risk conferred by additional chronic diseases was lower for heat stroke cases than for all heat illness cases and for any ED visits in general. This finding is somewhat difficult to interpret, possibly because heat stroke is a serious condition that frequently warrants admission in and of itself, and because comorbid conditions carry less weight in the admission decision calculus in such cases. But the fact that other diagnoses are more commonly listed first, suggesting greater concern about these other conditions, belies this interpretation. Alternatively, exposures may be different for those with heat stroke compared with other acute heat illnesses. Evaluation of the true relationship will likely require exposure data or perhaps other methods such as a chart review.

That some chronic diseases appear to confer greater risk is not surprising given the pathophysiology and social determinants of heat illness. The finding that chronic mental health disorders increase risk is well established, for instance (Page et al. 2012). The strongly increased risk associated with chronic hematologic disease, however, is a new finding, though an association between heat illness and sickle cell anemia (anemia is the most common chronic hematologic disease reported in the NEDS) is established (Smith et al. 2003). Although the association between chronic circulatory disease and death in the ED is established, its strength in our polytomous regression is remarkable and, like the other findings, requires confirmation and further study. Overall, our findings support the established public health tenet of importance of targeting prevention messages at patients with chronic conditions.

Our findings reinforce previous work regarding public health programming related to extreme heat. The large burden of heat illness in a working-age population may relate to occupational exposure though this warrants further study; regardless, prevention should target at-risk workers (Bernard and McGeehin 2004). The higher odds of adverse outcomes among elders suggest that efforts should target these populations as well (without creating a false sense of security in other groups), again consistent with prior findings (Luber and McGeehin 2008). We can also infer from our results that EDs should expect that those with the highest risk of dying in the ED may be the young, whereas increases in hospital admission are most likely to be for older patients with multiple chronic conditions.

These findings provide additional information for those interested in relating shifts in the frequency and severity of heat hazards, population susceptibility factors, and outcomes such as death in the ED and hospital admission. Our results may also have utility in projections of climate change impacts on heat morbidity or impacts on emergency services as well as more comprehensive estimates of costs associated with extreme heat events. To project such impacts, however, exposure–outcome associations will be needed, not just to characterize acute heat illness burdens but also to characterize increases in other heat-associated illness, as discussed above. These associations cannot be derived without linking exposure and outcome data.

Our findings speak to the strengths of the NEDS as a data set, including a large sample size, national representation, and availability of up to 15 ED diagnoses per visit. These strengths allow for a high level of statistical power in generating national estimates for small subpopulations and patients with multiple diagnoses, including chronic diseases. Comparison with other national ED data sources found that NEDS is among the best for detailed examination of diagnoses (Owens et al. 2010). Compared with the National Hospital Ambulatory Medical Care Survey (NHAMCS; http://www.cdc.gov/nchs/ahcd.htm), the other leading national ED data set, NEDS captures a higher number of diagnoses and a larger proportion of elderly patients (Owens et al. 2010). Compared with other databases for injury-related ED visits, which our outcomes include, the NEDS and the NEISS-AIP provide comparable estimates of injuries (though both provide lower injury estimates than NHAMCS) (Owens et al. 2010).

Despite these strengths, however, our study is subject to several limitations; the most important is the inability to link outcomes with exposure data. In the NEDS, ED visit date and location are restricted to protect patients’ and hospitals’ confidentiality. We used visit month as a proxy and excluded exposures of known man-made etiology. Although this approach captured 94% of heat illness cases, some could be unrelated to summertime heat exposure. Similarly, it is reasonable to note that the external validity of these findings depends on the study years 2006–2010 being representative not only of trends in non-NEDS states, which our analyses confirm, but also of recent temperature trends. Several of the years we studied were anomalously warm compared with climate normals, but this is in keeping with recent temperature trends (Hansen et al. 2012).

Some other potential limitations arising from the use of the NEDS data are also worth mentioning. The data included in the NEDS are based on the coding by providers and coders, and surveillance and research were not the intended use of these data. Moreover, admission diagnoses are also included for the subset of patients admitted to the hospital in the NEDS sample. These aspects of the survey’s design may introduce several biases into our results that bear mention.

Two potential biases may have resulted in overestimates related to severe heat illness. First, because there is no information on the inter-rater reliability of ED diagnoses of heat illness, it is possible that providers who treat patients with heat exhaustion are more likely to diagnose heat stroke than another acute heat illness if there are associated complications, even if there is no change in mental status. This could lead to an overestimate of heat stroke, though it is difficult to estimate the magnitude of its potential impact. Second, diagnoses are coded and arranged to maximize reimbursement for the care provided, not to facilitate epidemiologic research. This could introduce selection bias wherein severe illness is diagnosed more frequently. If present, this would also result in an overestimate of severe heat illness. We feel this is unlikely given that heat stroke was more often a secondary diagnosis, suggesting that the billing provider more often felt that heat stroke was secondary to another, more significant primary process.

Two other potential biases are worth noting. First, there may be have been detection bias from differential recognition of heat illness depending on perceptions of the weather (e.g., during the latter part of a declared heat wave). Conversely, there may have been underrecognition of heat illness during shorter periods of more routine weather. If present, such bias could result in over- or underestimation of heat illness, so it is difficult to assess its likely direction or magnitude. Second, providers probably did not code for all relevant chronic conditions. This would have resulted in an underestimate of the role of individual chronic disease categories in heat illness, particularly in treat-and-release visits, because those who were admitted presumably had a more exhaustive enumeration of comborbid and chronic conditions. To the extent that patients tend to have multiple chronic conditions, this underreporting may conversely have resulted in a diffuse overestimate of the observed associations with chronic disease burden. It is difficult to estimate the magnitude of these potential biases.

With these caveats in mind, we conclude that the NEDS provides a more comprehensive estimate of heat illness presentations to U.S. EDs than other nationally representative ED data sets and provides useful insights into the role of chronic conditions in the clinical course of heat-illness patients.

## Conclusion

Analysis of this nationally representative sample of ED visits for summertime acute heat illness demonstrates that its incidence was higher than anticipated, particularly in rural areas, among low-income communities, and among the uninsured. Most ED visits for heat illness were for less severe forms. Our analysis reveals patterns of heat illness similar to prior estimates but suggests a larger burden of disease. Population-based rates were highly correlated with average temperature anomalies in our 5-year sample, suggesting that ED visits for heat illness may be a reasonable surveillance indicator, though evaluation with a longer time series or more granular exposure information is warranted. Our findings provide new insight into dynamics surrounding heat stroke, which was diagnosed more frequently among older patients and those with multiple chronic conditions. Heat stroke cases had a high ED case fatality rate, and risk of death in the ED appears highest in the young, the poor, males, and Midwesterners. Chronic illness significantly increased risk of hospital admission or death in the ED, and the number of chronic conditions was associated with increased risk. The relative increase in risk is greater for all heat diagnoses than for heat stroke, highlighting the severity of this condition. Our findings reinforce previous work on heat preparedness, and may be useful in projecting heat morbidity associated with climate change. Future research could explore exposure–outcome associations by linking ED visits with temperature exposures and explore linkages between ED diagnoses and management with clinical outcomes and costs.

## Supplemental Material

(192 KB) PDF
