# Erratum: “Summertime Acute Heat Illness in U.S. Emergency Departments from 2006 through 2010: Analysis of a Nationally Representative Sample”

**DOI:** 10.1289/ehp.122-A293

**Published:** 2014-11-01

In the version of “Summertime Acute Heat Illness in U.S. Emergency Departments from 2006 through 2010: Analysis of a Nationally Representative Sample” by Hess et al. [Environ Health Perspect 122:1209–1215 (2014); http://dx.doi.org/10.1289/ehp.1306796] originally published online, low numbers of emergency department deaths (11–29) in Tables 2 and 3were mistakenly suppressed due to misinterpretation of the data-use agreement with the Agency for Healthcare Research and Quality (AHRQ). The tables have been updated in the final version of the article and are consistent with reporting requirements in the AHRQ data-use agreement (numbers of deaths ≤ 10 are suppressed).

The authors regret the error.

