# Supplementary material for: Summertime Acute Heat Illness in U.S. Emergency Departments from 2006 through 2010: Analysis of a Nationally Representative Sample
Source: Environ Health Perspect. 2014 Jun 17;122(11):1209–15. doi: 10.1289/ehp.1306796 (PMC4216158; doi:10.1289/ehp.1306796)
Supplement: (192 KB) PDF [file ehp.1306796.s001.508.pdf]

Environmental Health Perspectives doi:10.1289/ehp.1306796

Supplemental Material

Summertime Acute Heat Illness in U.S. Emergency Departments from 2006 through 2010: Analysis of a Nationally Representative Sample

Jeremy J. Hess, Shubhayu Saha, and George Luber

Table of Contents **Page**

Analysis of temperature maxima anomalies in NEDS versus non-NEDS states **2**

Table S1. States included in the NEDS sample by year 2

Table S2. Average annual maximum temperature anomalies in degrees Celsius for May-September inclusive compared with state-specific 1971-2000 baseline for NEDS and non-NEDS states, and annual rates of ED visits for heat illness per 100,000 population 3

Results of additional regression analyses **4**

Table S3. Polytomous regression odds ratios (95% CI) for hospital admission or death-in-the-ED versus treat-and-release among all acute heat illness cases 4

Table S4. Logistic regression odds ratios (95% CI) for hospital admission or death-in-the-ED versus treat-and-release among all heat stroke cases 5

Table S5. Logistic regression odds ratios (95% CI) for hospital admission or death-in-the-ED versus treat-and-release among all cases except heat stroke 6

Analysis of temperature maxima anomalies in NEDS versus non-NEDS states

The NEDS database is constructed with data provided by certain states, and national weights are constructed to produce nationally representative estimates. The states included in each year's sample are listed in Table S1.

Table S1. States included in the NEDS sample by year.

Year	States included in the NEDS
2010	AZ, CA, CT, FL, GA, HI, IA, IL, IN, KS, KY, MA, MD, MN, MO, NC, NE, NJ, NV, NY, OH, RI, SC, SD, TN, UT, VT, and WI (Added NV, Removed ME, NH)
2009	AZ, CA, CT, FL, GA, HI, IA, IL, IN, KS, KY, MA, MD, ME, MN, MO, NC, NE, NH, NJ, NY, OH, RI, SC, SD, TN, UT, VT, and WI (Added IL)
2008	AZ, CA, CT, FL, GA, HI, IA, IN, KS, KY, MA, MD, ME, MN, MO, NC, NE, NH, NJ, NY, OH, RI, SC, SD, TN, UT, VT, and WI (Added KY)
2007	AZ, CA, CT, FL, GA, HI, IA, IN, KS, MA, MD, ME, MN, MO, NC, NE, NH, NJ, NY, OH, RI, SC, SD, TN, UT, VT, and WI (Added NC, NY, RI)
2006	AZ, CA, CT, FL, GA, HI, IA, IN, KS, MA, MD, ME, MN, MO, NE, NH, NJ, OH, SC, SD, TN, UT, VT, and WI

If the distribution of temperature in states that contributed data to NEDS (NEDS states) were different than the states that did not provide the data (non-NEDS states), then national estimates of acute heat illness could not be nationally representative for our purpose. Therefore, we examined temperature distributions in the two groups of states to determine if there were significant differences annually. We obtained Average Daily Maximum Temperatures for May-September for each state for 2006-2010 from the National Climatic Data Center (http://www.ncdc.noaa.gov/cag/). For each state and year, we computed the difference between the annual average and the 30-year baseline average (1971-2000) to determine whether that state had an unusually hot or cold year. We then aggregated these differences for the two groups of states. We used the Shapiro-Wilk statistic to evaluate whether the yearly temperature differences among the two groups of states were normally distributed and then used t-tests to compare the temperature differences between the two groups. We found statistically significant differences only for 2009, as shown in Table S2 below.

Table S2. Average annual maximum temperature anomalies in degrees Celsius for May-September inclusive compared with state-specific 1971-2000 baseline for NEDS and Non-NEDS states, and annual rates of ED visits for heat illness per 100,000 population[a].

Year	Average anomaly, non-NEDS states	Average anomaly, NEDS states	ED heat illness visit rate
2006	0.90	0.69	23.30
2007	1.58	1.50	22.30
2008	-0.04	-0.04	18.10
2009[b]	0.16	-0.48	15.80
2010	1.8	2.11	27.90

[a]Population-based rates are presented in Table 2 of the main manuscript and repeated here for clarity. [b]The difference between the two groups of states was statistically different as indicated by t-test comparing equality of group means.

Results of additional regression analyses

Table S3. Polytomous regression odds ratios (95% CI) for hospital admission or death-in-the-ED versus treat-and-release among all acute heat illness cases.

	Hospital admission	Death in ED
**Age groups**		
0-17 yrs	0.38 (0.35,0.41)	2.56 (1.23,5.3)
18-45 yrs	0.42 (0.39,0.44)	0.93 (0.51,1.7)
45-65 yrs	0.57 (0.54,0.6)	0.87 (0.51,1.49)
65+ yrs (ref)	1	1
**Gender**		
Male	1.68 (1.63,1.74)	2.06 (1.46,2.9)
Female (ref)	1	1
**Urban-rural classification**		
Large metropolitan	1.33 (1.27,1.4)	1.55 (0.91,2.63)
Medium-small metropolitan	0.92 (0.87,0.97)	0.82 (0.47,1.42)
Micropolitan	0.87 (0.83,0.93)	1.28 (0.71,2.31)
Rural (ref)	1	1
**Health insurance**		
Medicare	1.01 (0.96,1.07)	1.01 (0.57,1.77)
Medicaid	0.91 (0.86,0.96)	2.26 (1.5,3.4)
Private	0.92 (0.88,0.96)	0.19 (0.11,0.35)
Uninsured (ref)	1	1
**Median income ZIP code**		
Top quartile	0.83 (0.8,0.86)	0.62 (0.44,0.88)
Quartile 3	0.78 (0.75,0.81)	0.53 (0.35,0.81)
Quartile 2	0.8 (0.76,0.84)	0.34 (0.19,0.59)
Lowest quartile (ref)	1	1
**Geographic regions**		
Midwest	1.12 (1.06,1.18)	3.53 (2,6.23)
South	0.87 (0.83,0.91)	0.79 (0.44,1.41)
West	0.83 (0.78,0.87)	0.38 (0.18,0.8)
Northeast (ref)	1	1
**Associated chronic conditions**		
Blood	11.9 (11.18,12.68)	1.63 (0.61,4.33)
Circulatory	2.42 (2.34,2.5)	21.87 (14.91,32.08)
Digestive	2.06 (1.95,2.17)	0.85 (0.37,1.95)
Endocrine	2.85 (2.76,2.94)	0.34 (0.21,0.54)
Genito-urinary	3.55 (3.33,3.79)	-
Mental	2.84 (2.75,2.92)	0.7 (0.47,1.03)
Muscular	2.47 (2.32,2.62)	2.33 (1.2,4.55)
Nervous	3.72 (3.54,3.9)	0.69 (0.28,1.69)
Respiratory	1.91 (1.82,1.99)	-
Cancer	2.83 (2.47,3.25)	3.66 (1.32,10.14)
No comorbid conditions (ref)	1	1

Table S4. Logistic regression odds ratios (95% CI) for hospital admission or death-in-the-ED versus treat-and-release among all heat stroke cases (ICD-9-CM 992.0) (N = 15759[a]).

Variable	OR (95% CI)
**Age groups**	
0-17 yrs	0.69 (0.57,0.84)
18-45 yrs	0.71 (0.6,0.83)
45-65 yrs	0.86 (0.74,1)
65+ yrs (ref)	1
**Urban-rural classification**	
Large metropolitan	2.47 (2.13,2.86)
Medium-small M=metropolitan	2.15 (1.85,2.49)
Micropolitan	1.33 (1.13,1.58)
Rural (ref)	1
**Health insurance**	
Medicare	1.49 (1.28,1.75)
Medicaid	0.96 (0.83,1.1)
Private	0.95 (0.85,1.07)
Uninsured (ref)	1
**Median income ZIP code**	
Top quartile	0.64 (0.56,0.73)
Quartile 3	0.59 (0.52,0.66)
Quartile 2	0.72 (0.65,0.8)
Lowest quartile (ref)	1
**Geographic regions**	
Midwest	0.61 (0.53,0.71)
South	0.94 (0.82,1.07)
West	1.01 (0.87,1.16)
Northeast (ref)	1
**Gender**	
Male	1.7 (1.55,1.87)
Female (ref)	1
**Associated chronic conditions**	
Blood	6.83 (5.71,8.17)
Circulatory	2.37 (2.15,2.6)
Digestive	1.51 (1.25,1.83)
Endocrine	2.47 (2.23,2.74)
Genito-urinary	2.03 (1.64,2.51)
Mental	2.4 (2.18,2.64)
Muscular	1.49 (1.23,1.82)
Nervous	4.44 (3.78,5.23)
Respiratory	1.93 (1.65,2.25)
Cancer	4.06 (2.5,6.6)
No comorbid conditions (ref)	1
**Year**	
2010	0.76 (0.67,0.86)
2009	0.63 (0.55,0.72)
2008	0.7 (0.62,0.8)
2007	0.87 (0.77,0.98)
2006	1

[a]The number of cases is smaller compared to Table 3 as some explanatory variables had missing variables.

Table S5. Logistic regression odds ratios (95% CI) for hospital admission or death-in-the-ED versus treat-and-release among all cases except heat stroke (ICD-9-CM 992.1-9) (N = 266984[a]).

Variable	OR (95% CI)
**Age groups**	
0-17 yrs	0.35 (0.32,0.38)
18-45 yrs	0.41 (0.39,0.44)
45-65 yrs	0.55 (0.52,0.58)
65+ yrs (ref)	1
**Urban-rural classification**	
Large metropolitan	1.24 (1.17,1.31)
Medium-small metropolitan	0.84 (0.8,0.89)
Micropolitan	0.87 (0.81,0.92)
Rural (ref)	1
**Health insurance**	
Medicare	0.95 (0.89,1)
Medicaid	0.86 (0.81,0.92)
Private	0.94 (0.9,0.98)
Uninsured (ref)	1
**Median zipcode income**	
Top quartile	0.79 (0.75,0.83)
Quartile 3	0.79 (0.76,0.83)
Quartile 2	0.85 (0.82,0.88)
Lowest quartile (ref)	1
**Geographic regions**	
Midwest	0.9 (0.85,0.96)
South	0.93 (0.88,0.98)
West	0.98 (0.92,1.05)
Northeast (ref)	1
**Gender**	
Male	1.63 (1.57,1.69)
Female (ref)	1
**Associated chronic conditions**	
Blood	9.34 (8.68,10.05)
Circulatory	2.47 (2.38,2.56)
Digestive	2.19 (2.06,2.32)
Endocrine	2.91 (2.8,3.01)
Genito-urinary	3.61 (3.36,3.88)
Mental	2.77 (2.68,2.87)
Muscular	2.56 (2.39,2.73)
Nervous	3.08 (2.91,3.25)
Respiratory	1.92 (1.82,2.02)
Cancer	2.54 (2.17,2.96)
No comorbid conditions (ref)	1
**Year**	
2010	0.73 (0.7,0.76)
2009	0.7 (0.66,0.74)
2008	0.81 (0.77,0.85)
2007	0.87 (0.83,0.91)
2006	1

[a]The number of cases is smaller compared to Table 3 as some explanatory variables had missing variables.
